# Potential for endocannabinoid system modulation in ocular pain and inflammation: filling the gaps in current pharmacological options

**DOI:** 10.1042/NS20170144

**Published:** 2018-11-02

**Authors:** J. Daniel Lafreniere, Melanie E.M. Kelly

**Affiliations:** 1 Department of Pharmacology, Dalhousie University, Halifax, NS, Canada; 2 Department of Ophthalmology and Visual Sciences, Dalhousie University, Halifax, NS, Canada; 3 Department of Anesthesia, Pain Management and Perioperative Medicine, Dalhousie University, Halifax, NS, Canada

**Keywords:** endocannabinoids, inflammation, neuropathic pain, ocular

## Abstract

Challenges in the management of ocular pain are an underappreciated topic. Currently available therapeutics lack both efficacy and clear guidelines for their use, with many also possessing unacceptable side effects. Promising novel agents would offer analgesic, anti-inflammatory, and possibly neuroprotective actions; have favorable ocular safety profiles; and show potential in managing neuropathic pain. Growing evidence supports a link between the endocannabinoid system (ECS) and a range of physiological and disease processes, notably those involving inflammation and pain. Both preclinical and clinical data suggest analgesic and anti-inflammatory actions of cannabinoids and ECS-modifying drugs in chronic pain conditions, including those of neuropathic origin. This review will examine existing evidence for the anatomical and physiological basis of ocular pain, specifically, ocular surface disease and the development of chronic ocular pain. The mechanism of action, efficacy, and limitations of currently available treatments will be discussed, and current knowledge related to ECS-modulation of ocular pain and inflammatory disease will be summarized. A perspective will be provided on the future directions of ECS research in terms of developing cannabinoid therapeutics for ocular pain.

## Introduction

**Figure 1:**
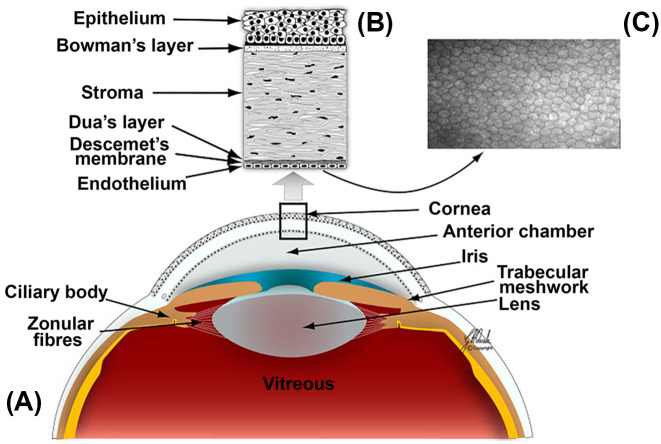
Overview of anterior ocular anatomy and corneal histology. Anterior ocular anatomy (**A**), with histologic cross-section of the cornea (**B**) and confocal microscopy of the corneal endothelium (**C**). Figure used with permission from [4].

The eye is a unique sensory organ comprised of both neural and non-neural tissues that facilitate vision through the collection and modification of light, followed by photoreceptor activation and subsequent neural transmission to vision centers of the brain. The anterior segment includes the highly innervated cornea, as well as the iris, lens and anterior chamber, the latter of which is filled with aqueous humor (Figure 1A). The posterior segment includes the retina, optic nerve, and the posterior chamber, which is filled with gel-like vitreous humor. The eye consists of an outermost fibrous sclera, covered by a thin membranous conjunctiva at its anterior aspect, with a vascular choroid layer below the sclera and superficial to the innermost retina—covering a large portion of the posterior chamber [1,2]. Six extraocular muscles act to delicately coordinate the voluntary and reflexive movements of the eye [3].

The blood–ocular barrier isolates the ocular environment from the systemic circulation, formed through both the blood–retina and blood–aqueous barriers [5]. The human cornea is avascular and thinnest centrally (0.5 mm), increasing in thickness peripherally [6]. Six layers comprise the cornea (Figure 1B), beginning with an outermost non-keratinized stratified squamous epithelium, below which lies a thin acellular layer (Bowman layer) followed by stroma that comprises 80–85% of the corneal thickness [6]. The corneal endothelium is in direct contact with the aqueous humor in the anterior chamber, representing the innermost layer of the cornea. Of note, the cornea is one of the most innervated tissues in the body [7], which imparts a unique sensitivity. In addition to the cornea’s ability to provide a refractive surface, and its barrier action, this sensitivity plays an important physiological role in tear film maintenance, where sensing evaporation triggers tear production [8]. Corneal innervation emanates from the nasociliary branch of the ophthalmic division of the trigeminal nerve that extends to a plexiform arrangement of nerves beneath the basal layer of the corneal epithelium [6]. The nature of this exposed and highly innervated system underlies the range of ocular pain pathologies.

## Mechanisms of ocular pain

Ocular pain that originates at ocular sites in the periphery is transmitted to sensory and emotional centers of the brain, forming the perception of pain. Specifically, ocular innervation originates from the ophthalmic division of the trigeminal nerve (cranial nerve V), with up to 450 free nerve endings of trigeminal sensory neurons in the epithelium of the cornea [9]. Nociceptors are the ‘noxious receptors’ and respond to a range of stimuli, including mediators of inflammation such as Substance P (SP), acetylcholine, bradykinin, or prostaglandins [10]. Those nociceptors that respond to multiple types of noxious stimuli are designated ‘polymodal’ [11]. As reported by Belmonte et al. [12], primary afferent nerve fibers in the cornea are either polymodal (70% of primary afferents), cold receptors (10%), or mechanoreceptors (20%). Cold receptors respond to low temperatures and may be involved with sensation of tear film evaporation [8], whereas mechanoreceptors respond to mechanical stimulation.

There are two main types of afferent neuronal fibers of nociceptors: Aδ- and C-fibers. Aδ fibers are myelinated and responsible for the transmission of noxious stimuli that leads to the perception of pain that is acute and localized. C-fibers are unmyelinated and responsible for the transmission of noxious information that results in a slow and diffuse pain [13]. Both Aδ- and C-fibers are present in the human cornea [14], with the majority being polymodal nociceptors [7]. When a nociceptor receives a stimulus of sufficient magnitude to surpass its activation threshold, neuronal membrane depolarization occurs [15]. This nociceptor depolarization occurs via several voltage-gated ion channels (sodium, calcium, and potassium), in addition to purinergic P2X families, acid-sensing ion channels, and members of the transient receptor potential (TRP) family of cation channels [16]. With sufficient nociceptors activated, this membrane depolarization generates action potentials in the primary afferent neurons, which synapse with second order neurons in the trigeminal brainstem complex before being transmitted to the higher centers responsible for emotional (amygdala, prefrontal cortex, periaqueductal gray, and hypothalamus) and sensory-discriminative (posterior thalamus, parabrachial nucleus, and insular cortex) aspects of the perception of pain [11,15,17–21].

**Figure 2:**
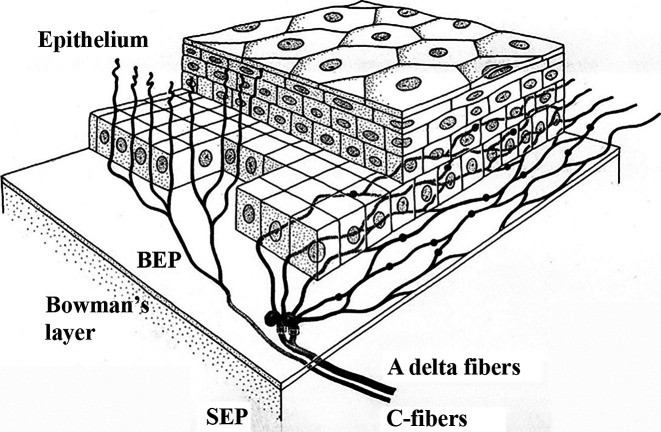
Depiction of human corneal epithelial innervation. Outlines stromal nerve bundles, including both Aδ and C-fibers, below Bowman’s layer in the SEP that enters the corneal epithelium via the basal lamina and forms the basal epithelial plexus, with Aδ fibers extending horizontally and C-fibers extending anteriorly. Figure used with permission from [23].

With regard to corneal innervation, the primary afferent fibers form bundles around the limbus, which leads to the generation of a limbal plexus [22]. As illustrated in Figure 2, both myelinated Aδ and the smaller diameter C-fibers originate from the subepithelial plexus (SEP) [23]. C-fibers extend anteriorly to the outermost layers of the corneal epithelium, while Aδ fibers branch dichotomously from the SEP, travelling anteriorly to Bowman’s membrane and then inferiorly to the basal epithelial cells [23].

Several key receptors present in corneal tissues contribute to the generation of pain signals. The TRP family of receptors, with notable members TRP vanilloid 1 (TRPV1) and TRP melastatin 8 (TRPM8), are cation channels present in the cornea that are implicated in sensory neurotransmission [24–32]. TRPM8, the so-called ‘cold receptor’, is the main receptor associated with cold somatosensation (temperatures < 25°C) but is also activated by menthol, icilin, and acetone [32–34]. Further, TRPM8 is proposed to play a physiological role in the sensation of tear film evaporation [8]. TRPV1 is the capsaicin-receptor, also activated by decreases in pH, temperatures above 43°C [35], a range of chemicals, hyperosmolarity, and mechanical stretch [36]. TRPV1 is known to modulate nociception, mediate innate immune responses and inflammation, as well as wound healing [37]. The activation of TRPV1 on nociceptive neurons leads to the generation of action potentials in addition to triggering the release of pain-inciting neuropeptides and inflammatory mediators [38]. In addition to the presence of TRPV1 throughout the human cornea (epithelial, stromal, and endothelial tissue) [29], TRPV1 is found on a range of peripheral nerve terminals as well as in the CNS [36]. Activation of TRPV1 through cross-talk with the cannabinoid 1 receptor (CB1R) has been reported, [30,39] and will be discussed in greater detail later in this review.

Other mediators of ocular pain include the vasoactive neuropeptides calcitonin gene-related peptide (CGRP) and SP, both known to modulate neurogenic inflammation in the eye [24,40,41]. SP and CGRP have been detected in sensory neurons in the cornea [9,24,42–46], and, following their release from activated nociceptive C-fibers [7,47], are associated with the transmission of pain, in addition to their pro-inflammatory actions [48,49]. As reported by Murata et al. [24], 37% of rat corneal neuronal somata in the ophthalmic division of the trigeminal nerve are TRPV1 positive (detected via immunofluorescence), with one third of these co-expressing substance P and the remainder co-expressing CGRP.

## Ocular disease and ocular pain

Pain is defined by the International Association for the Study of Pain as an ‘unpleasant sensory and emotional experience associated with actual or potential tissue damage, or described in terms of such damage’ [50]. Ocular pain has both diverse presentations and etiologies, with the potential to drastically alter quality of life, particularly when chronic [51]. Further, the management of chronic ocular pain can be challenging due to its complex origins, as well as the limitations of currently available therapies [52].

A range of pathologies can lead to acute or chronic ocular pain, which contributes to the marked burden of the condition. These include traumatic injury to the tissues of the eye or higher order nerves, infections (e.g. infectious keratitis or endophthalmitis), inflammatory conditions (such as uveitis), or iatrogenic causes (damage from surgery or infection secondary to invasive procedures) [20,53]. Systemic autoimmune disease such as diabetes can result in the development of chronic neuropathic pain [8,53].

Neuropathic pain results from central and/or peripheral nerve damage or sensitization. Patients with neuropathic pain may experience spontaneous pain, pain in response to a stimulus that does not normally elicit pain (i.e. allodynia), or an increased pain response resulting from a stimulus that normally elicits pain (i.e. hyperalgesia) [54,55]. In neuropathic pain syndrome, both an increased level of afferent nerve firing and an abnormal response to afferent stimuli are observed [56]. Spontaneous pain can originate from injured areas along the length of the nerve, such as nerve terminals or the dorsal root ganglion (DRG) [57], in addition to potentially originating from undamaged surrounding nerves that are activated via ‘cross-talk’ with damaged nerves [58].

Regarding the anterior ocular surface, trauma to the cornea (surgery, abrasions, foreign bodies, chemical burns etc.) or infections such as herpes simplex virus (HSV) keratitis can lead to nerve damage, with resultant pain. Conditions of the posterior segment, such as scleritis or idiopathic orbital inflammation, can lead to deep ocular pain that may be severe [59]. Also relating to the posterior segment, optic neuritis, characterized by inflammation of the optic nerve, may be painful (particularly with eye movement) [60].

Dry eye disease (DED) is among the most common ocular diseases [61] and is associated with chronic dry eye-like pain (DELP). Several comprehensive reviews discuss the relation between DED and DELP in detail, such as Rosenthal et al. and Galor et al. [8,62]. Under normal physiologic conditions, the corneal surface is covered by a thin tear film, composed of a mucinous layer that originates from goblet cells in the conjunctiva, an aqueous layer superficial to the mucinous layer, and an outermost lipid layer [6]. The tear film plays a key barrier role and through the interface generated with the cornea, contributes significantly to ocular refractive power [6]. It is interesting that decreases in the parameters of tear production are not well associated with DELP [8,63,64]. Similarly, there is not a strong association between disorders of the meibomian glands and DELP—where meibomian glands normally supply the lipid component of the tear film [8,52]. Further, DELP exhibits characteristics of neuropathic sensitization of the cornea [8]. Several studies have suggested that TRPM8 receptors on the ocular surface are involved in sensing tear evaporation [65,66], suggesting an intricate role of TRPM8 in the physiological maintenance of a tear film. Significantly decreased tear production has been observed in TRPM8 knockout (*Trpm8^−/−^*) mice [65]. Through recording nerve terminal impulse activity in the mouse eye, this group additionally identified loss of cold responsiveness in TRPM8 knockouts.

Inflammatory pain is a form of nociceptive pain. The process of inflammation can, however, itself lead to the development of neuropathic pain [67–71]. This inflammatory neuronal damage can occur directly, such as in autoimmune neuropathy, or indirectly, such as from nerve compression, which may result in ischemic nerve injury. Neural sensitization and lowered pain thresholds occur with tissue damage, resulting in the release of a range of inflammatory mediators such as cytokines, bradykinin, SP and prostaglandins, which further contribute to neuronal sensitivity [56]. Ocular inflammation is a double-edged sword in that it plays a key role in antimicrobial defenses and wound healing, yet at the same time can be central in pathological processes that pose a threat to vision. The eye is uniquely isolated from the systemic immune system (‘immune-privileged’) via various barriers (physical, immunosuppressive factors, and active immune modulation) [72,73], which confers protection from the potentially damaging effects of immune infiltration [74]. At the same time, dendritic cells and resident macrophages in various ocular tissues, such as the cornea and choroid, act as sentinels of the innate immune system (an initial non-specific response) [75,76].

### Current therapies for ocular pain and limitations

Commonly used topical agents in the management of ocular pain include anesthetics, non-steroidal anti-inflammatory drugs (NSAIDs), and corticosteroids [77–79]. Neuropathic pain is treated with systemic agents aimed at modulating pain-signaling pathways, in order to increase nociceptor pain thresholds and decrease signaling. These include the gabapentinoids (‘GABAergics’), tricyclic antidepressants, and opioids [53]. Acute pain treatment is often initiated alongside disease-specific treatments, such as surgical management or antibiotics. Current pharmacological options for the treatment of neuropathic pain fall short due to inadequate efficacy and/or unacceptable side effects [80,81].

Topical ocular anesthetics (such as tetracaine or proparacaine) are sodium channel blockers, widely used in clinical practice, and can result in immediate and complete alleviation of pain that originates from the ocular surface. This efficacy is, however, contrasted by limits on duration of use, where continued application is well documented to lead to corneal melt [82]—an ulcerative process associated with breakdown of corneal epithelium and stroma [77].

Corticosteroids (such as prednisolone or dexamethasone) act as potent anti-inflammatory agents, through up-regulation of anti-inflammatory protein transcription and blockade of the translocation of pro-inflammatory transcription factors. Topical steroids remain key tools in decreasing ocular inflammation, which can be vision-saving [83]; however, their extended use is associated with significant side effects including cataract, glaucoma, delayed ocular healing, or secondary infections resulting from ocular immunosuppression [84]. Systemic steroids or other immunosuppressive agents are used in addition to NSAIDs in severe inflammatory conditions such as scleritis [85].

NSAIDs inhibit cyclooxygenase enzymes (COX-1 and COX-2), preventing the formation of certain eicosanoids, notably prostaglandins—which are inflammatory and nociceptive [86,87]. Topical ocular NSAIDs (such as nepafenac or ketorolac) are generally associated with a less significant toxicity profile than are corticosteroids [77]. There remain, however, concerns relating to ocular NSAID safety, particularly in the context of long-term use. The ocular toxicity of NSAIDs is associated with delayed healing [88] and ranges from epithelial erosion and corneal thinning, to corneal melt [77]. Systemic NSAIDs such as ibuprofen or naproxen are associated with inhibition of COX-1 mediated gastroprotective actions, resulting in increased risk of gastric ulceration and bleeds, in addition to increased cardiovascular risks and nephrotoxicity [89–91]. Topical and systemic NSAIDs are generally not considered to be options for the treatment of neuropathic pain. Acetaminophen, which acts differently than do NSAIDs, may play a limited role in pain management, particularly as an adjunctive agent, potentially acting through peripheral and central COX inhibition, in addition to central mechanisms including modulation of descending serotonergic pathways and ECS modulation [92].

Neuropathic pain generally requires systemic pain-modulating strategies, usually beginning with gabapentinoid agents (e.g. pregabalin, gabapentin) or certain antidepressants—namely the tricyclic antidepressants (TCAs) (e.g. amitriptyline, nortriptyline) or serotonin norepinephrine reuptake inhibitors (SNRIs) (e.g. venlafaxine, duloxetine) as first-line agents [93]. Second-line agents include tramadol—a weak opioid agonist and SNRI, and classical opioid receptor agonists (e.g. hydromorphone, fentanyl). The mechanisms of the gabapentinoids in neuropathic pain are not well defined; however, their blockade of voltage-gated calcium channels (VGCCs) on primary afferent neurons is believed to play a central role [94,95], which appears to be in-line with up-regulation of a specific VGCC subunit (α2δ) in the setting of nerve injury [96]. Use of a combination of these agents may achieve only moderate reductions in pain, and may carry a heavy side-effect burden, such as dizziness and somnolence for gabapentinoids, weight gain, daytime sedation and other anticholinergic effects for TCAs, or constipation, nausea, sweating and potential for clinically significant respiratory depression with the use of opioids [93].

The use of systemic cannabinoids is promising in managing chronic non-cancer pain, particularly when neuropathic in nature [97,98]. Cannabinoid-mediated analgesia arises from a range of peripheral and central mechanisms, including through modulation of descending inhibitory pain pathways, central neuronal modulation, neuroprotective mechanisms, and inhibition of prostaglandin synthesis [99]. Evidence supporting use of systemic cannabinoids for pain is reflected by their incorporation into the Canadian Pain Society guidelines as third line agents for chronic neuropathic pain [93]. There is, however, a clear need for further large-scale controlled clinical trials in order to establish both safety and efficacy of systemic cannabinoids [100–103]. With respect to the ocular use of cannabinoids and small molecular drugs that target the ECS, several recent studies have indicated that ECS-modifying drugs may be useful in the treatment of both ocular pain and inflammation, with options for local ocular delivery offering advantages over systemic cannabinoids, such as decreased systemic bioavailability and potentially improved target-site concentrations. The following sections will review current knowledge of the ECS, the presence of components of the ECS in the eye, and existing evidence for ECS modulation of ocular pain.

## Overview of the endocannabinoid system

The ECS is a complex biological regulatory system, conserved among vertebrates, which regulates both systemic and CNS functions that include immune response, pain, neuronal activity, in addition to metabolic and cardiovascular functions [104–109]. Growing evidence also suggests that the ECS plays an important role in a range of disease processes [110]. The characterization of an endogenous cannabinoid system stemmed from the isolation of the psychoactive cannabinoid, THC (Δ^9^-tetrahydrocannabinol), from cannabis in 1964 by Gaioni and Mechoulam [111]. Identification of THC and the synthesis of synthetic cannabinoid ligands heralded the discovery and cloning of receptors for cannabinoids and facilitated identification of endogenous ligands. The two main endogenous bioactive lipids of the ECS, termed endocannabinoids, are anadamide (AEA) [112] and 2-arachidonoylglycerol (2-AG) [113]. Together with their respective biosynthetic and degradative enzymes, endocannabinoids and cannabinoid receptors constitute the ECS (Figure 1) [114].

The cannabinoid receptors include the CB1R—found in the CNS and ubiquitously expressed in systemic organs and tissues, and the cannabinoid type 2 receptor (CB2R)—highly localized to cells of the immune system. The first discovered cannabinoid receptor, CB1R, was cloned from rat brain cDNA in 1990 [115], and subsequent identification of CB2R was based on sequence homology with CB1R (44% amino acid homology is shared) [116]. Both CB1R and CB2R are G-protein coupled receptors (GPCRs), activated endogenously by endocannabinoids, the most well documented of which are AEA and 2-AG [117,118]. The endocannabinoids also bind to non-cannabinoid receptors, including TRPV1, G-protein coupled receptor 55 (GPR55) (Figure 1), GPR18 and peroxisome proliferator-activated receptors α, β/δ, and γ [119]. All tissues of the body, which have been examined to date, contain endocannabinoids [120].

**Figure 3:**
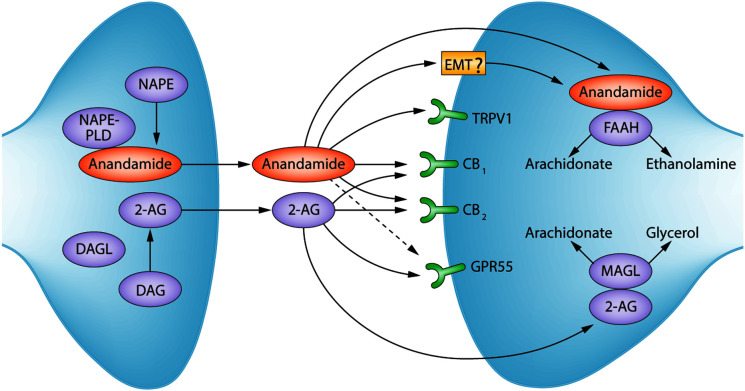
Overview of the ECS. Highlights key endocannabinoids, cannabinoid receptors, and biosynthetic and degradative enzymes. NAPE: *N*-acyl-phosphatidylethanolamine; NAPE-PLD: *N*-acyl-phosphatidylethanolamine-specific phospholipase D; 2-AG: 2-arachidonoylglycerol; DAG: diacylglycerol; DAGL: diacylglycerol lipase; EMT: endocannabinoid membrane transporter – ? is to denote controversy surrounding the characterization of an EMT [132–137]. TRPV1: transient receptor potential cation channel subfamily V member 1; CB1: cannabinoid receptor 1; CB2: cannabinoid receptor 2; GPR55: G protein-coupled receptor 55; FAAH: fatty acid amide hydrolase; MAGL: monoacylglycerol lipase. Figure modified with permission from [114].

**Figure 4:**
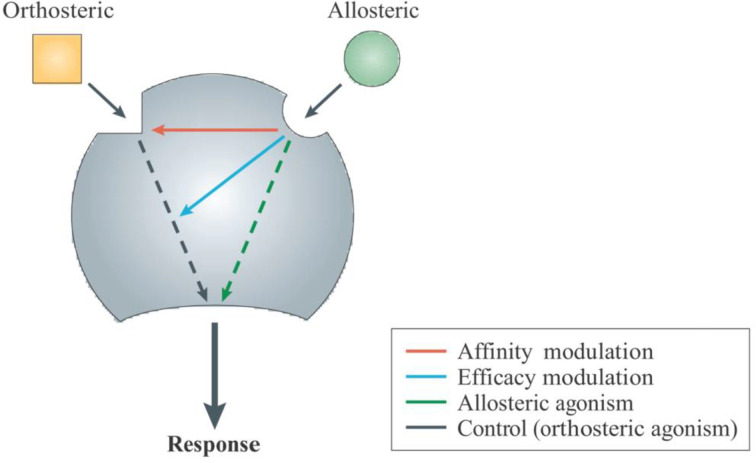
Mechanistic summary of allosteric receptor modulation. Figure used with permissions from [138].

Endocannabinoids are generated ‘on-demand’ via enzymatic catabolism of membrane lipids [121] (Figure 3). Specifically, AEA is formed through the enzymatic action of phospholipase D (NAPE-PLD) on phospholipid N-arachidonoyl phospahtidylethanolamine (NAPE) [120–122]. Synthesis of 2-AG begins with the generation of 1,2-diacylglycerol (DAG) via phospholipase C, where DAG is then converted to 2-AG via 1,2-diacylglycerol lipase (DAG lipase) [120,123,124]. Degradation of endocannabinoids also occurs through enzymatic action, and the localization of specific degradative enzymes dictates the duration of action of endocannabinoids (Figure 4) [108,120,125]. AEA is converted via hydrolysis to arachidonic acid and ethanolamine by the enzyme fatty acid amyl hydrolase (FAAH), and 2-AG is converted via hydrolysis to arachidonic acid and glycerol through the actions of monoacylglycerol (MAGL), and to a lesser extent α–β hydrolase domain protein 6 (ABHD-6) and ABHD-12 [120,126–130]. Both 2-AG and AEA act as substrates for arachidonic acid-metabolizing enzymes such as lipoxygenase enzyme subtypes or COX-2, where AEA also acts as a substrate for certain cytochrome P450 oxidase enzymes [131].

The well-known psychoactive actions of the cannabis plant arise largely from THC-mediated activation of CNS CB1Rs, which are coupled to Gαi protein-signaling pathways including adenylyl cyclase (AC), mitogen-activated protein kinase (MAPK), and cyclic adenosine monophosphate (cAMP) [108,118]. CB1R activation results in decreased synaptic neurotransmitter release mediated through presynaptic voltage-gated Ca^2+^ channel-inhibition [121]. The presence of CB2R on immune cells spans various populations, listed here in order of descending levels of receptor expression: natural killer cells, monocytes, polymorphonuclear leukocytes, and CD4^+^ and CD8^+^ lymphocytes [139]. In addition to expression on immune cells of the periphery, CB2R is also found in the CNS on microglia cells, glia and some neurons [140,141]. Activation of CB2R is not associated with CB1R-like behavioral effects, but in common with CB1R, activation of CB2R is associated with Gαi protein-coupled signaling pathways, inhibition of AC and decreases in cAMP levels as well as activation of MAPK pathways [108,117,142,143]. Growing evidence links CB2R activation with anti-inflammatory actions [108,144–148]. Further, increased levels of both endocannabinoids and CB2R expression have been observed in the setting of injury, indicative of the receptor’s involvement in endogenous protective mechanisms [149,150].

### Ocular ECS and potential for modulation

No ECS-modulating agents are currently approved for ocular use. There is, however, a growing body of evidence indicating potential for ECS-modulation in the treatment of a range of ocular conditions. Both CB1R and CB2R have been identified in various tissues of the human eye, including the cornea (CB1R) [151], ciliary body and ciliary process, trabecular meshwork, retina, and iris [151–153] as well as in immune cells present in ocular tissues (CB2R) [139,154–157]. Both endocannabinoids (2-AG and AEA) and the enzymes involved in their synthesis and degradation have been identified in various tissues in human eyes, including the cornea, iris, retina, choroid, and ciliary body [157,158–165].

CB1R is of interest as an analgesic strategy, and the activation of CB2R is of particular interest in terms of its anti-inflammatory effects [108]. Alternate ECS-modulation strategies, such as allosteric modulation [166–170] or degradative enzyme inhibitors [171] may additionally be valuable in achieving site-specific cannabinoid actions and/or serve to elicit cannabinoid receptor effects while minimizing the side-effects that are associated with chronic administration of potent orthosteric agonists (i.e. the generation of tachyphylaxis) [157,164]. Ocular ECS-modulation is a promising potential target for a number of painful ocular diseases, and the development of appropriate preclinical models will be an important next step in assessing safety and efficacy of cannabinoids for ocular disease, particularly in terms of their long-term use.

#### ECS actions on ocular inflammation

Inflammation in the eye can lead to acute or chronic pain, the development of neuropathy, or contribute to vision loss [20,21]. A range of leukocyte populations express CB2Rs and studies in animal models have generated promising data, which indicates anti-inflammatory effects of CB2R-activation [144,146,147]. Though significant levels of CB2R have not been detected in corneal tissue under physiological conditions [172], CB2R-modulation is a promising target for corneal disease as the immune cells that express CB2R are recruited to the eye during ocular inflammation. Additionally, up-regulation of CB2R on resident immune cells and glia after injury may also be relevant for development of CB2R-selective drugs [173].

A commonly used animal model of ocular inflammation involves intraocular administration of endotoxin (lipopolysaccharide, LPS), termed endotoxin-induced uveitis (EIU). Administration of LPS results in an innate immune-mediated pan-ocular inflammation that mimics pathophysiological characteristics of anterior uveitis, an inflammatory condition of the uvea (encompassing the iris, ciliary body, and choroid), which is accompanied by pain and photophobia [146,147,174]. An EIU study examining cytokine and chemokine levels, as well as leukocyte–endothelial adherence in the microvasculature of the rat iris, as parameters of inflammation, compared ocular topical treatment with a CB2R-selective cannabinoid agonist to topical NSAID (nepafenac) and corticosteroids (prednisolone and dexamethasone) [146]. The CB2R-agonist resulted in decreased parameters of inflammation at 6 h, where, interestingly, similar anti-inflammatory actions were not observed with NSAID or corticosteroids. It is also noteworthy that CB1R activation may not be advantageous during EIU, as suggested by one study in rabbits that found AEA-induced increases in parameters of ocular inflammation and damage, which were attenuated with CB1R blockade (via AM251) [175].

Experimental autoimmune uveoretinitis is an antibody/adjuvant-triggered adaptive immune-mediated inflammation that induces pathological changes in-line with uveoretinitis [73,144]. Using this model, a selective CB2R-agonist (JWH 133) was found to suppress the disease progression and led to several immunosuppressive actions, including decreased cytokine/chemokine production, as well as decreased leukocyte rolling and infiltration in vasculature of the inflamed retina when delivered systemically [144]. The authors propose that these effects may result from CB2R-mediated inhibition of autoreactive T-cell activation, in addition to effects on leukocyte recruitment.

A recent study by Szczesniak et al. demonstrated anti-inflammatory actions of cannabinoids in proliferative vitreoretinopathy (PVR), an inflammatory condition that develops secondary to ocular trauma, or iatrogenic causes—commonly procedures used to correct retinal detachment [176,179]. In a mouse PVR model using intravitreal (IVT) dispase (to cleave the retinal basement membrane), treatment with a cannabidiol (CBD) analog, HU308, which selectively activates CB2R [177,178], led to decreased parameters of inflammation and improved histopathology [179]. This study by Szczesniak et al. found worsened histopathology and increased parameters of inflammation with CB2R-knockout or pharmacological blockade of CB2R (via AM630), indicating a tissue-protective and anti-inflammatory role of CB2R. Further, beneficial effects of HU308 (anti-inflammatory and improved histopathology) were absent when HU308 was administered to mice pretreated with a CB2R antagonist (AM630), indicating these actions of HU308 were CB2R-mediated.

#### ECS actions on ocular pain

There are limited yet promising lines of evidence indicating the potential for ECS-modulation in the management of ocular pain. A study in rats that elicited ocular pain (via ocular surface administration of mustard oil or a CO_2_ gas puff) found decreased neuronal activity of those corneal nerves that synapse in a specific medullary transition region, the trigeminal interpolaris/caudalis (Vi/Vc) following treatment with a non-selective CB1R/CB2R agonist (WIN55212-2) [180]. The authors related this action to the Vi/Vc’s role in corneal reflexes and anterior eye homeostasis.

Recently published data in a mouse model of corneal hyperalgesia found that three topically applied cannabinoids (Δ^8^THC, CBD, and HU-308) decreased corneal inflammation and corneal hyperalgesia, as determined by the evaluation of corneal neutrophil infiltration and behavioral testing, respectively [181]. All tested cannabinoids had both analgesic and anti-inflammatory actions in wild-type mice, with a CB1R-antagonist, AM251, blocking both the analgesic and anti-inflammatory effects of Δ^8^THC, suggesting that the effects of Δ^8^THC are mediated by CB1R activation [181]. The actions of the phytocannabinoid, CBD, however, did not result from CB1R or CB2R modulation (confirmed using AM251 and CB2R-knockout mice). Interestingly, concurrent administration of a 5-hydroxytryptamine subtype 1a (5-HT_1A_) receptor antagonist (WAY100635) ablated the effects of CBD. Both the analgesic and anti-inflammatory actions of the CB2-selective CBD analog, HU308, were absent in CB2R-knockout mice, supporting contributions of CB2R-mediated corneal anti-inflammatory and antinociceptive effects in this model [181].

The ocular actions of cannabinoids may involve non-cannabinoid receptors, as seen with potential CBD anti-inflammatory and analgesic actions that are mediated through 5-HT_1A_ [181]. Further, the TRPV1 receptor has been found to co-localize with CB1R in both mouse corneal epithelium as well as human corneal epithelial cells, implying that CB1R may play an important role in modulating corneal TRPV1 activity [27,30]. Yang et al. [30] further identified reductions in both inflammatory infiltration of the corneal stroma and scarring in an injury model, following treatment with WIN55212-2. The authors proposed that these actions were through CB1R–TRPV1 interactions and involved TRPV1 receptor desensitization. Similarly, findings were reported by McDowell et al. [182] in rat DRG neurons where sensitization of TRPV1 (via nerve growth factor) was inhibited by CB1R-activation (via arachidonoyl-2’-chloroethylamide). Understanding the interaction between CB1R with TRPV1 may be valuable in developing therapeutics for ocular pain, as both receptors are present in ocular tissues and involved in ocular pain transmission.

#### Novel approaches to modulate the ocular ECS: cannabinoid receptor allosteric modulation and enzyme-inhibitors as indirect ECS agonists

As reviewed by Cairns et al., the potential for ECS modulation by exogenous endocannabinoids maybe limited due to their short duration of action [164,183,184]. In addition, ligands that bind to the orthosteric site in order to activate cannabinoid receptors are associated with psychoactive effects (via CB1R), dependence, and the development of tachyphylaxis when administered repeatedly [166,169,170,185]. Together, these underline the need for further exploration of novel ECS-modulatory strategies, including the use of allosteric ligands or enzyme inhibitors.

Figure 4 illustrates allosteric ligand modulation of receptors. Through binding ‘allosteric’ sites on the receptor—sites distinct from the orthosteric site, allosteric ligands can increase (positive allosteric modulators, PAMs) or decrease (negative allosteric modulators, NAMs) a receptor’s interaction with orthosteric agonists, thus altering ligand affinity, efficacy, potency, and/or dissociation [167,168,138]. In addition to PAMs and NAMs, there are ‘neutral’ allosteric ligands, which compete with ligands for allosteric site binding while not eliciting effects on response to orthosteric site ligand binding [167,168,138]. Cumulative evidence indicates multiple allosteric sites on GPCRs, which expands the range of modulatory strategies [168,169].

Allosteric ligands have several unique properties that contribute to their potential clinical utility. One such property is the tendency of allosteric sites to be more distinct from one another (via demonstrating less conservation between receptor subtypes) than are orthosteric sites [167,170]. This has been demonstrated for CB1R, where allosteric sites are believed to be located in variable regions [186–188], as compared with orthosteric site(s) found in more conserved regions [170,189–191]. Congrève et al. [168] suggest that an evolutionary pressure toward conservation of orthosteric sites, for the preservation of endogenous ligand signaling, is likely responsible and absent in the case of allosteric sites. This may lead to less off-target side effects from allosteric ligands compared with orthosteric ligands [192].

Biased ligands are able to elicit a shift toward a receptor conformation that favors specific signaling pathways [169]. Although biased signaling is not unique to allosteric ligands, this, along with the distinct nature of allosteric sites provides opportunities to develop novel therapeutics with improved specificity and reduced side effects [169,193–197]. Allosteric modulation of cannabinoid receptors is a complex and novel area for discovery. Illustrating this complexity is data indicating a range of possible outcomes of modulation. Studies have demonstrated the ability of allosteric modulators to possess both PAM and NAM actions. An example of this is with an allosteric modulator possessing PAM activity for CB1R orthosteric agonist binding, with concurrent NAM activity on CB1R-mediated inhibition of cAMP [169,198,199].

Tachyphylaxis represents an adaptive mechanism, characterized at the cellular level for cannabinoids by cannabinoid receptor desensitization and down-regulation, which, along with CB1R-mediated psychotropic actions, limits the clinical applications of CB1R orthosteric agonists [200]. Allosteric modulators have the potential to increase cannabinoid receptor activity without contributing to desensitization [164,166]. Novel strategies include allosteric modulation of endogenous endocannabinoid binding, and PAM-mediated potentiation of sub-threshold levels of exogenous agonists. With respect to CB1R, these strategies may reduce the development of tachyphylaxis following repeat cannabinoid receptor agonist treatment [201]. This likely has significant potential in developing novel ECS-modulating strategies, particularly for frequent and/or long-term use, such as in uveitis, glaucoma, or chronic ocular pain conditions. In support of this approach, a recent publication by Cairns et al. reported that topical application of a novel CB1R allosteric modulator, GAT229 [202], was able to reduce intraocular pressure (IOP) in a mouse model of glaucoma. Additionally, when combined with a CB1R agonist, WIN55212-2, at a subthreshold dose for reducing IOP, GAT229 was able to increase the efficacy and duration of action for WIN55212-2’s ocular hypotensive actions [203].

A second novel approach to ECS-modulation is through the inhibition of mechanisms by which endocannabinoid action is terminated. Enzyme inhibitors exert cannabimimetic actions through inhibiting the degradative enzymes responsible for endocannabinoid hydrolysis (predominant mechanism) or oxygenation [171,204]. As with allosteric modulators, an enzyme inhibitor strategy may result in site-specific effects. This is likely a result of increased endocannabinoid synthesis at the site of tissue injury [205]. Generally, endocannabinoids are short-lived, undergoing both rapid enzymatic degradation and uptake from the extracellular space [171,206,207]. Potential enzyme inhibitors may target FAAH, increasing the levels of AEA, and/or MAGL, ABHD-6, and ABHD-12—to increase the levels of 2-AG [171]. Toczek et al. [171] provide a comprehensive review of the ECS enzyme inhibitors examined in preclinical studies and clinical trials.

To date, endocannabinoid enzyme inhibitors have not been examined in the context of ocular pain and inflammation. That being said, there is considerable data indicating that they possess analgesic actions in neuropathic pain models [171]. The most work to-date has focused on FAAH inhibition and indicates the antinociceptive [208–213], neuroprotective [214,215] and analgesic actions [216] including in inflammatory pain models [217,218].

It is interesting that a peripherally restricted FAAH inhibitor, URB937, was shown to be antinociceptive in both inflammatory and peripheral nerve injury pain models, with both effects lost through CB1R blockade [219]. As suggested by the authors, these results indicate peripheral AEA actions via CB1R, which prevent signal transmission to higher centers in the CNS. This peripherally restricted FAAH inhibitor has been further shown to possess antinociceptive action in a migraine model (via systemic nitroglycerin) in rats [220]. There remains, however, a level of complexity to the effects of FAAH in pain. Carey et al. identified a pro-nociceptive phenotype (nocifensive behavior and hypersensitivities to mechanical and heat stimuli) with capsaicin challenge in mice with genetic knockout of FAAH, while the mice concurrently demonstrated an analgesic phenotype in response to models of inflammatory nociception [221]. Both TRPV1- and CB1R-antagonists ablated this pro-nociceptive phenotype, and the authors acknowledged the gaps in knowledge surrounding the range of active lipid signaling molecules that are metabolized by FAAH, with these data suggesting potential limitations of FAAH inhibition based on pain type [221]. Further, it is of value to acknowledge that attempts to examine systemic FAAH-inhibition in the setting of chronic pain have generated conflicting findings [222,223], including a drug candidate (PF-04457845, Pfizer) that was dropped after clearing Phase II clinical trials due to poor efficacy [224]. Several new FAAH inhibitors with improved efficacy are in development; however, this development was placed on hold pending an investigation of the pharmaceutical company Bial’s agent, 10-2474, which led to the development of severe neurological disorders during a phase I clinical trial in 5 of 6 subjects, with 1 death [225]. This complication is, however, suggested to have resulted from off-target and non-ECS related actions [226].

Enzyme inhibitors targeting MAGL have also generated promising data; however, these inhibitors have been associated with behavioral effects indicative of CB1R activation in the CNS [227]. While FAAH inhibitors have been reported to generate analgesia in preclinical models, these drugs, unlike MAGL inhibitors, have not generally been associated with behavioral effects [228,229]. These differences between FAAH- and MAGL- inhibitor action may in part be due to the full versus partial agonist actions of 2-AG and AEA, respectively [230].

JZL184 acts as a MAGL-specific inhibitor [229], which allows differentiation from FAAH-inhibition mediated effects [231], and has been shown to produce analgesia in various pain assays [229,232–237]. In a mouse model of orofacial neuropathic pain, induced via injury of the inferior orbital nerve, JZL184 reduced pain (increased threshold to mechanical stimulation) 2 h following administration [231].

Another MAGL-selective inhibitor, KML29, has reported anti-inflammatory and antinociceptive action [238]. Crowe et al. assessed low-dose KML29 in combination with a low-dose of the established neuropathic pain agent, gabapentin, in a mouse model of neuropathic pain (chronic constriction injury) [239]. The authors highlight this strategy as a method to limit side effect profiles of the individual agents, finding that treatment resulted in additive attenuation of mechanical allodynia (reduced with CB1R antagonist treatment) and synergistic reduction of cold allodynia (no effect with CB1R blockade) [239]. Along a similar line of investigation, Crowe et al. [240] assessed the combination of the NSAID diclofenac with JZL184, finding additive attenuation of cold allodynia, as well as synergistic reduction of mechanical allodynia, which was blocked with CB1R antagonist treatment. Also examining KLM29, Miller et al. [241] observed an ocular hypotensive effect in a mouse model, while JZL184 did not have an effect on IOP.

While endocannabinoid enzyme inhibitors have not been specifically examined in models of ocular inflammation and pain, Slusar et al. [214] examined the effect of FAAH inhibition with URB567 on the survival of retinal ganglion cells (RGCs), in a rat optic nerve axotomy model. The study demonstrated an increased survival of RGCs with URB597 treatment, an effect ablated following CB1R but not CB2R antagonist treatment. This group also identified significant increases in retinal AEA and decreased AEA metabolite levels following URB597 treatment.

Creative approaches to ECS modulation in the treatment of ocular pain and inflammation offer unique advantages over exogenous orthosteric modulation. A significant body of research indicates analgesic and antinociceptive actions of enzyme inhibitors, including of FAAH and MAGL, in neuropathic and other pain models. The actions of enzyme inhibitors specifically in the setting of ocular pathology have demonstrated promising data indicating neuroprotective and IOP-lowering actions, although examination of selective endocannabinoid enzyme inhibitors in relevant animal models of ocular inflammation and pain is now required in order to fully access the utility of this class of ECS-modulators. As the cellular uptake of endocannabinoids plays a role in termination of action, strategies to inhibit this process may also be valuable when attempting to prolong endocannabinoid action.

## Conclusion

There is a definite need for novel pharmacological approaches to the management of ocular pain. In particular, ocular neuropathic pain is difficult to manage and may originate from non-ocular sites in the periphery and/or centrally. The ECS is present ubiquitously through the body, including a range of ocular tissues, and represents a promising target in the treatment of several physiological and pathophysiologic processes in the eye including, but not limited to, pain, inflammation, and neuronal damage. Modulating CB2R has immunomodulatory potential, whereas the activation of CB1R appears to contribute to both centrally and peripherally mediated analgesia. Novel drug strategies, namely the use of cannabinoid receptor allosteric modulators, biased ligands or through the inhibition of enzymes that degrade endocannabinoids, may be valuable targets in developing drugs that circumvent the limitations of orthosteric agonists at the cannabinoid receptors. Furthermore, ocular topical or regional administration of cannabinoids is a promising strategy for delivery. For cannabinoids administered via systemic or topical/regional routes alike, safety and efficacy must be further examined, particularly in the context of long-term use.

## Abbreviations

2-AG: 2-arachidonoylglycerol
5-HT_1A_: 5-hydroxytryptamine receptor 1A
ABHD: α–β hydrolase domain
AC: adenylyl cyclase
AEA: anadamide
cAMP: cyclic adenosine monophosphate
CB1R: cannabinoid 1 receptor
CB2R: cannabinoid 2 receptor
cDNA: complementary deoxyribonucleic acid
CGRP: calcitonin gene related peptide
CNS: central nervous system
COX-1 and -2: cyclooxygenase 1 and cyclooxygenase 2
DAG: diacylglycerol
DAGL: diacylglycerol lipase
DED: dry eye disease
DELP: dry eye-like pain
DRG: dorsal root ganglion
ECS: endocannabinoid system
EIU: endotoxin-induced uveitis
EMT: endocannabinoid membrane transporter
FAAH: fatty acid amide hydrolase
GPCR: G protein-coupled receptor
GPR55: G protein-coupled receptor 55
HSV: herpes simplex virus
IOP: intraocular pressure
LPS: lipopolysaccharide
MAGL: monoacylglycerol lipase
MAPK: mitogen-activated protein kinase
NAM: negative allosteric modulator
NAPE: *N*-acyl-phosphatidylethanolamine
NAPE-PLD: *N*-acyl-phosphatidylethanolamine-specific phospholipase D
NSAID: non-steroidal anti-inflammatory drug
PAM: positive allosteric modulator
PVR: proliferative vitreoretinopathy
RGC: retinal ganglion cell
SEP: subepithelial plexus
SNRI: serotonin norepinephrine reuptake inhibitor
SP: substance P
TCA: tricyclic antidepressant
THC: tetrahydrocannabinol
TRP: transient receptor potential
TRPV1: transient receptor potential vanilloid 1
TRPM8: transient receptor potential melastatin 8
VGCC: voltage-gated calcium channel
Vi/Vc: trigeminal interpolaris/caudalis
